# Pediatric reflex syncope: updated insights and future directions

**DOI:** 10.1007/s00431-026-06786-w

**Published:** 2026-03-05

**Authors:** Vincenzo Russo, Angelo Comune, Giangiacomo Di Nardo, Giovanni Maria Di Marco, Gabriella Gaudieri, Erika Parente, Alfredo Caturano, Andrea Antonio Papa, Anna Rago, Maria Giovanna Russo, Gerardo Nigro

**Affiliations:** 1 Cardiology and Syncope Unit, Department of Medical Translational Sciences, University of Campania “Luigi Vanvitelli,” Monaldi Hospital, Naples, Italy; 2 Department of Pediatric Cardiology, Santobono-Pausilipon Childrens Hospital, Naples, Italy; 3 Pediatric Cardiology Unit, Department of Medical Translational Sciences, University of Campania “Luigi Vanvitelli”, Monaldi Hospital, Naples, Italy; 4 Department of Human Sciences and Promotion of the Quality of Life, San Raffaele Roma Open University, Rome, 00166 Italy

**Keywords:** Pediatric syncope, Vasovagal syncope, Head-up tilt test, Implantable loop recorder, Fludrocortisone, Midodrine, Cardioneuroablation

## Abstract

Reflex syncope is the most frequent cause of transient loss of consciousness in the pediatric population. A structured diagnostic approach based on clinical evaluation and 12-lead ECG is mandatory to exclude the cardiac causes of syncope. Additional cardiac investigations, such as echocardiography, a stress test, or 24H Holter ECG monitoring, are needed in case of suspected cardiac syncope at initial evaluation. Cardiovascular autonomic function assessment, including ambulatory blood pressure monitoring and a tilt test, is useful for phenotyping syncope (hypotensive or bradycardic mechanism). In case of unexplained syncope after a comprehensive evaluation and high-risk criteria, an implantable loop recorder is indicated. The management is primarily based on reassurance, education, hydration, increased salt intake, and counter-pressure maneuvers. Pharmacological therapies and intervention strategies may be considered for patients with recurrent or disabling forms that are not responsive to lifestyle modifications.

*Conclusion*: Reflex syncope in the pediatric population should be managed through a structured diagnostic pathway focused on excluding cardiac causes and guiding mechanism-based treatment. Education and lifestyle measures remain the cornerstone of management, while pharmacological or invasive strategies should be reserved for selected patients with recurrent or disabling symptoms.

**Table:** 

**What is Known:** • *Reflex syncope is the most common cause of transient loss of consciousness in children and adolescents, and initial evaluation should rely on careful history taking, physical examination, and a 12-lead ECG to exclude cardiac causes.* • *Most pediatric reflex syncope can be managed conservatively through education and reassurance, together with adequate hydration, increased salt intake, and physical counter-pressure maneuvers*.	
**What is New:** • *This review proposes a structured stepwise diagnostic pathway that starts with clinical evaluation and ECG and escalates only when cardiac syncope is suspected or the presentation is high-risk.* • *It emphasizes the role of brief cardiovascular autonomic assessment (ambulatory blood pressure monitoring and tilt testing) to distinguish hypotensive from bradycardic mechanisms and guide individualized management.*

**What is Known:**

• *Reflex syncope is the most common cause of transient loss of consciousness in children and adolescents, and initial evaluation should rely on careful history taking, physical examination, and a 12-lead ECG to exclude cardiac causes.*

• *Most pediatric reflex syncope can be managed conservatively through education and reassurance, together with adequate hydration, increased salt intake, and physical counter-pressure maneuvers*.

**What is New:**

• *This review proposes a structured stepwise diagnostic pathway that starts with clinical evaluation and ECG and escalates only when cardiac syncope is suspected or the presentation is high-risk.*

• *It emphasizes the role of brief cardiovascular autonomic assessment (ambulatory blood pressure monitoring and tilt testing) to distinguish hypotensive from bradycardic mechanisms and guide individualized management.*

## Introduction

Syncope is defined as a transient loss of consciousness (TLOC) with loss of postural tone due to brief global cerebral hypoperfusion, characterized by rapid onset, short duration, and complete spontaneous recovery [1]. Syncope is a frequent clinical event among children and adolescents; indeed, up to 20% of them experience at least one syncopal episode before 18 years [2]. Reflex syncope, the most common and benign cause of syncope, is due to a temporary failure of the autonomic nervous system to maintain blood pressure and/or heart rate. In particular, the sudden triggering of abnormal autonomic reflex leads to vasodilatation and/or bradycardia [1]. However, a structured, stepwise diagnostic approach is required to exclude potentially life-threatening cardiac disorders [3]. This narrative review summarizes the current evidence on the diagnostic evaluation and therapeutic approach to pediatric patients with reflex syncope.

## Epidemiology

Syncope is a common finding in pediatric populations, although its incidence and etiology vary by age. Reflex syncope is uncommon in early childhood, where cardiac or neurological causes are proportionally more frequent (2–6%) [4]; however, breath-holding spells (BHS), occurring in about 5% of children between 6 and 18 months, may be associated with syncope [5]. Syncope prevalence rises with age, from 2–3% in preschool children to 8% in school-aged children and nearly 30% in adolescents [6], peaking between 15 and 19 years and showing a clear female predominance [7]. It accounts for 1–3% of pediatric emergency visits [8], while cardiac etiologies occur in less than 1%, confirming the overall benign nature of pediatric syncope [9].

## Differentiating between syncope and seizure

Reflex syncope may occasionally present with brief, irregular jerking movements that can mimic an epileptic seizure, making a careful differential diagnosis essential. More than 20 rhythmic jerks strongly favor an epileptic origin, while syncopal jerks are fewer, asynchronous, and of shorter duration [4]. Lateral tongue biting is highly specific for epileptic seizures, whereas incontinence, although frequent during seizures, is not discriminative since it may also occur in syncope. Laboratory markers may aid differentiation: creatine kinase and lactate levels usually remain normal after syncope but often rise after seizures [2]. Recovery characteristics provide additional diagnostic clues: in epilepsy, return of consciousness is typically slow and followed by a prolonged postictal confusion, while recovery from vasovagal syncope is rapid and complete [4]. Epilepsy and syncope may occasionally provoke one another, since seizures can induce secondary syncope, as in “ictal asystole.”

## Initial clinical evaluation

The initial clinical evaluation of pediatric patients with syncope aims to exclude underlying cardiac disease and to confirm the reflex origin of the event. The structured, stepwise approach includes careful history-taking, physical examination, blood pressure measurement, and a 12-lead electrocardiogram (ECG) [1, 10, 11]. The detailed history remains the cornerstone of diagnosis and allows accurate classification in most pediatric cases [12]. History-taking should specifically explore the context and posture at onset, the presence of prodromal symptoms, and the recovery phase, as these elements are crucial to distinguish reflex syncope from cardiac or neurological causes [12]. Reflex syncope is typically triggered by prolonged standing, heat exposure, emotional stress, sight of blood, or pain. In contrast, episodes occurring during exertion or in the supine position are more suggestive of cardiac etiology. Episodes occurring during exertion are more suggestive of cardiac causes, such as structural or arrhythmic heart disease [1, 4, 8, 10, 11]. In contrast, events occurring immediately after exercise usually reflect a benign reflex mechanism related to abrupt withdrawal of sympathetic tone and reduced venous return [13]. Most reflex syncopal events are preceded by prodromal symptoms such as lightheadedness, blurred vision, nausea, sweating, pallor, palpitations, tinnitus (buzzing, roaring, ringing), muffled hearing, and subjective sensation of warmth or cold. The absence of prodromes does not exclude reflex syncope; however, it should raise concern for arrhythmic or structural heart disease [1, 4, 8, 10, 12]. Recovery is typically rapid and complete in reflex syncope, unlike the postictal confusion or prolonged disorientation often observed after seizures or cardiac syncope [10, 12].

In adults, careful history-taking, physical examination, and electrocardiogram allow identification of the likely cause of syncope in approximately 60–70% of cases [14, 15]. In children, the diagnostic yield of the first clinical evaluation is limited by communication barriers and reliance on witness accounts, particularly regarding prodromal symptoms, especially when history taking is performed by healthcare personnel with limited experience in pediatric patients [12].

The physical examination should include careful cardiac auscultation and palpation of peripheral pulses to detect murmurs, gallops, or pulse deficits suggestive of structural heart disease. Systolic ejection murmur is suggestive of aortic or pulmonary stenosis, according to the auscultation site (other than innocent/benign murmurs, atrial septal defects, and branch pulmonary artery stenosis). Weak femoral pulses and/or radial-femoral pulse delay suggest aortic coarctation. Blood pressure should be measured in supine and standing positions to assess orthostatic changes [16] and, when aortic coarctation is suspected, in both arms and a leg to detect inter-limb pressure gradients [17].

The 12-lead ECG allows the exclusion of primary electrical diseases and structural abnormalities presenting with syncope. ECG findings such as a long- or short-corrected QT interval, Brugada-like pattern, pre-excited QRS complexes, atrioventricular block, left bundle branch block, epsilon waves, or signs of ventricular hypertrophy should prompt referral for further cardiac evaluation [1, 4, 10].

## Additional diagnostic testing

The prescription of additional diagnostic tests should be based on initial clinical findings; they should not be used as routine screening [1]. Echocardiography is indicated for diagnosis and risk stratification in patients with suspected structural heart disease [1, 10]. When performed without clinical suspicion, echocardiography has no additional diagnostic value in pediatric syncope [18]. A 24-h Holter ECG should be considered in patients with frequent syncope episodes (≥ 1 per week) when an arrhythmic cause is suspected, particularly in the presence of palpitations, syncope during exertion, structural heart disease, a family history of sudden cardiac or ECG findings suggestive of an arrhythmic cause [1]. In the absence of clinical suspicion of arrhythmic syncope, its diagnostic yield is 1–2%; conversely, among pediatric patients with exertional syncope or a family history of sudden cardiac death (SCD), its diagnostic yield increases to 16% [19]. In patients with events that occur more frequently than 24 h but less frequently than monthly, event monitors or external loop recorders can be helpful. Exercise testing should be performed in patients who have experienced episodes of syncope during exertion. The diagnosis of reflex syncope is confirmed when syncope is reproduced immediately after exercise in the presence of severe hypotension [1]. Among pediatric patients with exertional syncope, mid-stride syncope is more likely to result in a cardiac diagnosis; among these, catecholaminergic polymorphic ventricular tachycardia was the primary etiology of exertional syncope [20]. Among adults with reflex syncope, 24-h ambulatory blood pressure monitoring (ABPM24H) has recently been included in the evaluation of patients with suspected reflex syncope to assess hypotensive profiles [21] or systolic blood pressure drops [22]. In this setting, ABPM can identify significant hypotensive episodes defined as at least one daytime SBP drop < 90 mmHg, (two SBP drops < 90 mmHg if the 24 h mean SBP < 125 mmHg) or at least two daytime drops < 100 mmHg. These cut-off values predict hypotensive susceptibility with limited sensitivity (29–40%) but high specificity (83–95%), suggesting a low probability of hypotensive susceptibility among patients without SBP drops on ABPM [22]. Among children, a standardized definition of a hypotensive profile is currently lacking. Giordano et al. [23], using normative data from Soergel et al. [24], applied an operational criterion defining hypotension as mean systolic or diastolic BP values below the 50th percentile for age, sex, and height. In their cohort, including 146 children (mean age 11.7 ± 7.1 years; 82 males) with unexplained syncope, 133 (91%) showed a low blood pressure pattern at 24 h-ABPM, and 72 (54%) had a positive tilt test response.

## Head-up tilt test

The head-up tilt test (HUTT) is a non-invasive diagnostic examination used to assess the cardiovascular response to postural changes and to induce, in a controlled setting, vasovagal syncope or other disorders of orthostatic adaptation [25]. HUTT should be performed in all pediatric patients with unexplained syncope at initial evaluation or in those with severe recurrent reflex syncope in order to identify the underlying mechanism and to guide the therapy. The nitroglycerin-potentiated HUTT achieves the highest diagnostic performance among available protocols, with an overall positivity rate of 74.9%, ranging from 51.5% to 81.6% among pediatric patients with non-classical (absence of prodromes or specific triggers) and classical presentation, respectively. The specificity is about 81.3% [26], maintaining an excellent safety profile and reproducibility across both adult and pediatric populations [27–30]. HUTT presents a major limitation: the tilt-induced reflex often fails to mirror spontaneous attacks, as bradyarrhythmias and asystolic pauses occur more frequently during real-life events than in a laboratory setting.

**Fig. 1:**
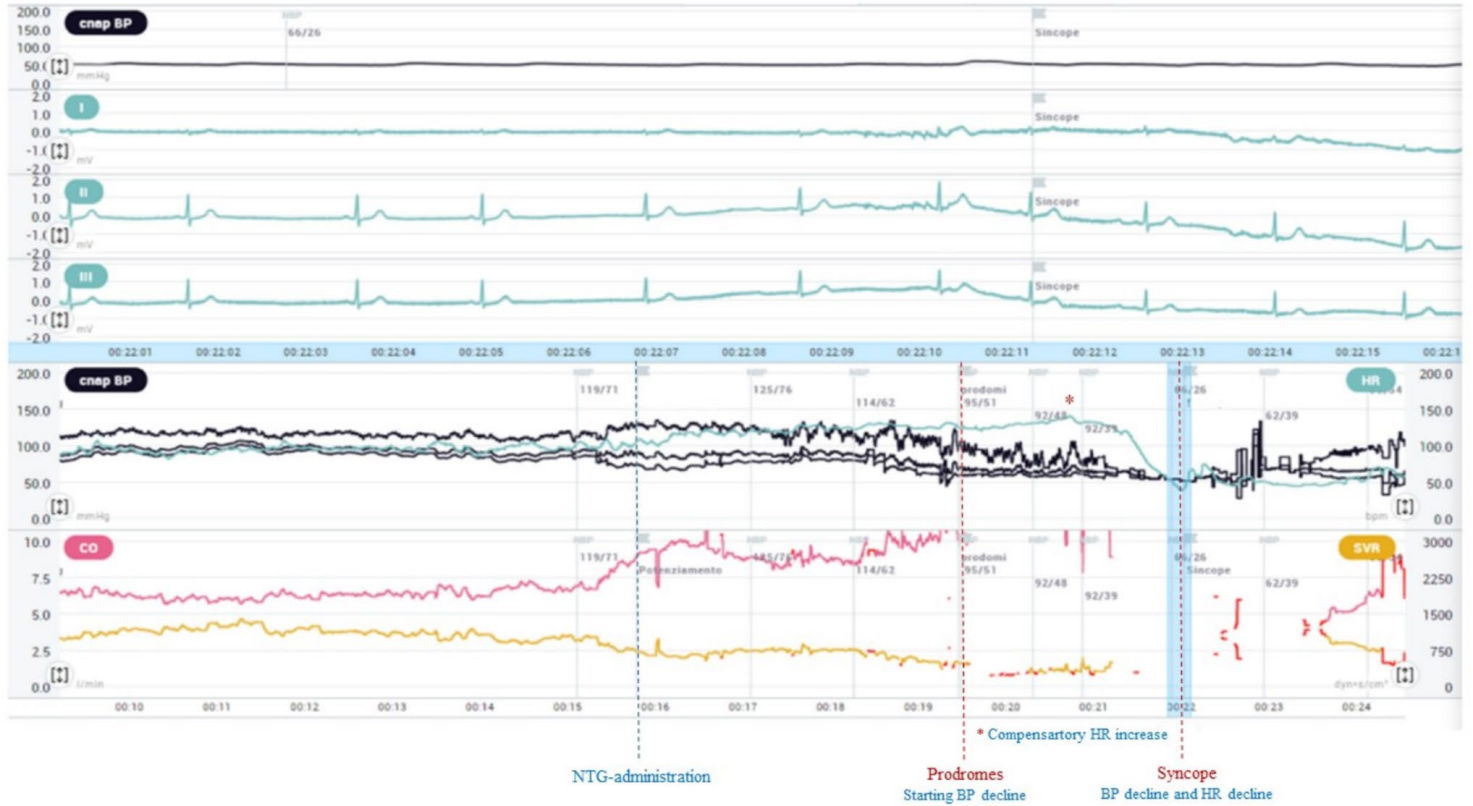
Non-invasive beat-to-beat blood pressure and ECG monitoring during tilt test in a pediatric patient

Recently, a randomized trial including 554 adult patients with suspect vasovagal syncope showed that a short NTG potentiated tilt test protocol (so-called Fast Italian Protocol) has the same diagnostic value as the traditional NTG potentiated protocol, with no significant difference in the distribution of hemodynamic responses. The Fast Italian protocol includes a supine pre-tilt phase of 5 min without venous cannulation, a passive tilt phase of 10 min at 70°, and, in case of negativity, a sublingual NTG spray at a fixed dose of 300 µg, followed by an active phase of 10 min. [31]. Figure 1 shows a case of tilt test with beat-to-beat blood pressure and ECG monitoring positive for mixed syncope.

Following nitroglycerin administration (dotted blue line), a progressive decline in systolic blood pressure is observed (black line), leading to prodromes (first dotted red line), initially counterbalanced by a baroreflex-mediated increase in heart rate (red asterisk). When the vagal reflex develops, an abrupt bradycardia and accentuated vasodepression lead to mixed syncope (second dotted red line).

## Implantable loop recorder

An implantable loop recorder (ILR) is a subcutaneous device that enables long-term, continuous ECG monitoring with both automatic and patient-activated recording of cardiac rhythm [32]. ILR implantation is recommended in patients with unexplained syncope when the initial diagnostic work-up is inconclusive and high-risk features are present [1]. Moreover, it may be useful when the prolonged external monitoring (e.g., 30-day loop recorders) fails to capture events due to infrequent episodes, poor patient compliance, or intolerance to external leads. Among pediatric patients, the family history of premature sudden cardiac death (< 40 years), the clinical features of the episode (syncope occurring during exertion, without prodromes, while supine or during sleep, or preceded by chest pain or palpitations), or some electrocardiographic abnormalities are considered high risk features [1, 32]. ILR implantation may also be considered when the differential diagnosis of transient loss of consciousness remains uncertain and a neurological or a neuropsychiatric disease is suspected [32]. In selected pediatric patients with recurrent, unexplained episodes mimicking seizures or psychogenic events, ILR monitoring can document the temporal relationship between symptoms and cardiac rhythm, helping to differentiate vasovagal syncope from epileptic seizures or revealing ictal arrhythmias associated with epileptic activity [33].

**Fig. 2:**
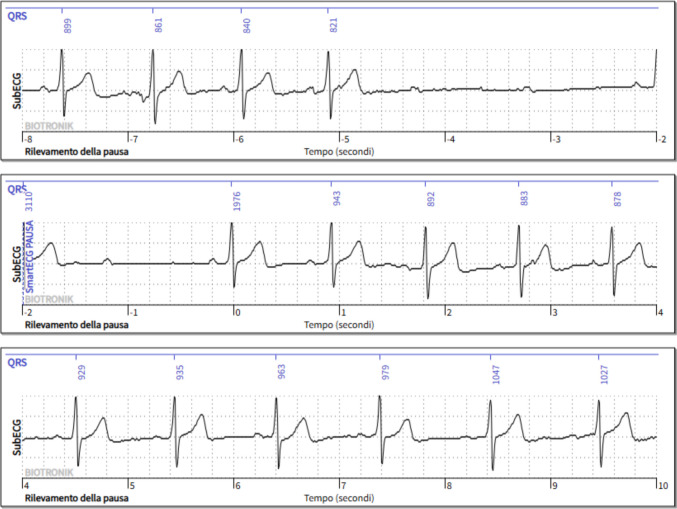
Asystole due to paroxysmal AV block detected by an implantable loop recorder

The diagnostic yield of ILRs in patients with unexplained syncope, seizure-like episodes, or infrequent palpitations after an inconclusive evaluation is approximately 3%, mainly due to the detection of bradyarrhythmias or intermittent atrioventricular block, with a median time to diagnosis of about 6 months [34, 35]. Data from large adult cohorts indicate that integrating remote monitoring technologies further enhances the clinical utility of ILRs, enabling faster detection of relevant arrhythmias, reducing time to diagnosis, and optimizing healthcare resource use compared with conventional in-office follow-up [36]. Figure 2 shows an ILR monitoring positive for symptomatic asystole due to paroxysmal atrioventricular (AV) block.

## Management

### Non-pharmacological strategies

The management of reflex syncope aims to reduce recurrence, prevent injury, and improve quality of life, with a primarily non-pharmacologic approach. All patients and their parents must be reassured about the benign nature of reflex syncope and taught to recognize the prodromal symptomatology.

Physical counterpressure maneuvers (leg crossing, squatting, handgrip, arm tensing, glute clenching) effectively abort impending syncope [37].

Adequate hydration (500 ml water in addition to daily requirements) and salt intake (≈5–6 g NaCl/day) reduce recurrence in pediatric cohorts [1, 38]. Tilt or orthostatic training may improve orthostatic tolerance but offers no additional benefit beyond structured self-care [37]. Regular aerobic exercise improves autonomic regulation and orthostatic tolerance and may help reduce the recurrence of reflex syncope in children and adolescents [39]. Attention to environmental factors—such as avoiding heat exposure, prolonged standing, and crowded or poorly ventilated settings—is also essential to prevent symptom recurrence and promote long-term stability [40].

### Pharmacological strategies

Among pediatric patients with recurrent or disabling reflex syncope unresponsive to lifestyle modification or counter-pressure maneuvers, pharmacologic therapy may be considered [10, 41]. The two most used agents are fludrocortisone (0.1–0.2 mg once daily) and midodrine hydrochloride (2.5–10 mg three times daily at 4-h intervals) [42]. Fludrocortisone, a synthetic mineralocorticoid, acts by promoting renal sodium retention and plasma volume expansion, thereby enhancing venous return and orthostatic tolerance while reducing susceptibility to hypotensive episodes [43]. Midodrine hydrochloride, through peripheral α-adrenergic stimulation, increases vascular tone and counteracts venous pooling, providing additional support in patients with refractory or hypotensive forms [44]. In adults, both fludrocortisone and midodrine reduce hypotensive episodes on ambulatory monitoring [45]; across them, fludrocortisone achieved the most consistent hemodynamic improvement. An average 24-h systolic BP increase of 7 mmHg was associated with a nearly 60% reduction in daytime hypotensive episodes, while a ≥ 13 mmHg rise was associated with fewer syncope recurrences [46]. The use of selective serotonin reuptake inhibitors (SSRIs) in vasovagal syncope is supported by randomized trials suggesting a potential role in reducing recurrence [47]. Their effectiveness in cardioinhibitory syncope with asystole remains anecdotal but may be explained by chronic central downregulation of serotonin receptors, which reduces excessive vagal efferent activation implicated in reflex bradycardia and asystole [48].

### Interventional strategies

Among pediatric patients with recurrent syncope unresponsive to lifestyle measures, counter-pressure maneuvers, or pharmacologic therapy, and evidence of asystole at a tilt table test or ILR, cardioneuroablation (CNA) may be considered in highly selected cases, although evidence in pediatric randomized trials is currently lacking. CNA is an interventional, catheter-based strategy consisting of endocardial radiofrequency ablation of atrial ganglionated plexi, mainly around the sinus node region, with the aim of attenuating parasympathetic input [49]. Emerging evidence supports its efficacy in recurrent cardioinhibitory syncope refractory to conventional therapy [50]. Pediatric experience is limited to isolated cases, including a 12-year-old with symptomatic sinus bradycardia successfully treated by anatomically guided CNA and symptom-free at follow-up [51]. Permanent pacing is not indicated for reflex syncope in children, even in the presence of documented asystole [1, 32]. However, closed-loop stimulation (CLS) is an advanced dual-chamber pacing algorithm that continuously monitors myocardial contractility via intracardiac impedance [52]. By enabling early detection of autonomic changes, CLS shows an additive effect on dual-chamber pacing in maintaining cardiac output and preventing syncopal recurrences among adults over 40 years with recurrent cardioinhibitory syncope and tilt-induced asystole [53–56].

Tables 1 and 2 show differences in the management of reflex syncope between pediatric and adult patients.

**Table 1:** Differences in management of reflex syncope between pediatric and adult patients

	Children	Adult
Clinical history taking	Limited by communication barriers and reliance on witness accounts	Usually easy to take
Office and home blood pressure	Uncertain low cut-off values	Known cut-off values
Standing BP (standing test)	OH difficult to ascertain	High diagnostic value of OH
ECG	High diagnostic yield	
Echocardiography	Low diagnostic yield in the absence of suggestive clinical or electrocardiographic findings	
24-h Holter ECG	Low yield with no arrhythmic suspicion; ~ 16% diagnostic yield in exertional syncope or family history of SCD	Low diagnostic yield
24-h ABPM	Uncertain diagnostic value No validated cut-off available	Diagnosis of hypotensive phenotype Validated cut-offs for hypotension and BP drops predictive of reflex syncope Established diagnostic utility
Stress test	Indicated in case of exertional syncope	
Tilt test (NTG protocol)	Rarely indicated Sensitivity of 51.5% among patients with non-classical presentation	Diagnostic yield is well established
Carotid sinus massage	Not indicated	Recommended when syncope is uncertain after the initial evaluation and for phenotyping
Implantable loop recorder	High diagnostic yield in unexplained syncope	
Therapeutic approach	Non-pharmacological (first line) Pharmacological (selected cases)	Non-pharmacological Managing comorbidities Deprescribing hypotensive therapies Pharmacological (selected cases)
Cardiac pacing	Not indicated	Indicated in documentation asystolic reflex syncope in selected adults aged > 40 years
Cardioneuroablation (CNA)	Potentially may be indicated in documented asystolic reflex syncope, but data are minimal (only case reports)	Increased evidence of documented asystolic reflex syncope in selected adults

**Table 2:** Gaps in evidence regarding reflex syncope in pediatric patients

**24-h ABPM**	No validated cut-off for defining hypotensive phenotype
Tilt testing	Uncertainty regarding the optimal dosage of NTG stressor Limited evidence on the use of the Italian FAST Protocol
Pharmacological therapy	Lack of controlled pediatric trials on fludrocortisone or midodrine Insufficient data on long-term safety and optimal dosing
Cardioneuroablation	Only case reports; no pediatric randomized studies

## Digital tools, wearable devices, and artificial intelligence in syncope.

Wearable devices are increasingly adopted in pediatric cardiology because of their non-invasive nature and high acceptability [57]. Modern smartwatches provide continuous photoplethysmographic monitoring and user-initiated single-lead ECG recordings, enabling documentation of heart-rate abnormalities and arrhythmias during daily activities. Consumer wearables have been shown to play a relevant role in arrhythmia diagnosis and surveillance in children, including cases in which conventional ambulatory monitoring was non-diagnostic [58]. However, smartwatch-derived tracings may be affected by motion artifacts and algorithmic misclassification, potentially leading to false or misleading diagnoses [59].

The increasing availability of continuous physiological data from wearable devices provides a natural framework for the application of artificial intelligence (AI) and machine-learning (ML) techniques in syncope. Supervised learning models use labelled clinical data to predict outcomes, whereas unsupervised approaches analyze unlabelled datasets to identify previously unrecognized patient phenotypes; deep-learning architectures further enable direct analysis of raw biosignals, such as ECGs, by automatically extracting non-linear features [60, 61]. In adult populations, ML-based tools have demonstrated high diagnostic performance in distinguishing syncope from other causes of transient loss of consciousness (random-forest sensitivity for syncope 100%, overall accuracy 86%) [62] and in predicting short-term serious outcomes (neural-network sensitivity 100%, specificity 79%) [63]. Deep-learning models applied to large ECG–echocardiographic datasets have also shown robust performance (AUC 0.86–0.89) in identifying structural heart disease potentially relevant to syncope mechanisms [64].

From an implementation perspective, the clinical value of AI appears particularly relevant in remote-monitoring settings, where continuous data acquisition is associated with a high burden of false-positive alerts. AI-based algorithms may improve signal interpretation, reduce false-positive transmissions, and decrease physician workload while preserving diagnostic sensitivity, thereby enhancing the efficiency and sustainability of digital monitoring pathways [65]. Overall, current evidence supports an augmented-intelligence paradigm in which AI complements clinical judgement; however, most available data derive from adult cohorts, and dedicated validation in pediatric syncope remains necessary [66].

## Conclusions

Reflex syncope is the leading cause of transient loss of consciousness in children and adolescents. The diagnosis is mainly led by anamnesis and clinical evaluation. The electrocardiogram is mandatory to exclude suspected cardiac syncope. ABPM24H and Tilt Testing provide information about the clinical phenotype (vasodepressor and cardioinhibitory) of reflex syncope. ILR implantation is indicated when syncope remained unexplained after a comprehensive evaluation and high-risk features are present. Management is mainly non-pharmacological, while pharmacologic therapy and interventional approaches may be considered in the case of recurrent, unpredictable syncope.

## Data Availability

No datasets were generated or analysed during the current study.
